# SEMA3B is associated with disease activity and infliximab response in IBD patients but does not contribute to the development of intestinal inflammation *in vivo*

**DOI:** 10.3389/fimmu.2026.1677130

**Published:** 2026-02-04

**Authors:** Laura Arosa, Beatriz Malvar-Fernández, José Antúnez-López, Samuel García, Javier Conde-Aranda

**Affiliations:** 1 Molecular and Cellular Gastroenterology Group, Health Research Institute of Santiago de Compostela (IDIS), Santiago de Compostela, Spain; 2 Rheumatology & Immuno-mediated Diseases Research Group (IRIDIS), Galicia Sur Health Research Institute (IIS Galicia Sur), SERGAS-UVIGO, Vigo, Spain; 3 Rheumatology Department, University Hospital of Vigo, Vigo, Spain; 4 Department of Pathology, University Clinical Hospital of Santiago de Compostela, Santiago de Compostela, Spain

**Keywords:** anti-TNF, biomaker, colitis, inflammatory bowel disease, semaphorin

## Abstract

**Background:**

Inflammatory bowel disease (IBD) is a chronic inflammatory disorder with increasing worldwide incidence and prevalence. While current treatment options alleviate part of the socioeconomic burden of this disease, new biomarkers and safer therapeutic approaches are needed to combat intestinal inflammation. Class-3 semaphorins (sema3A-3G) have emerged as important regulators of some biological functions underlying inflammation. For instance, SEMA3B protects against tissue damage in arthritis. However, the association of this protein with UC and its involvement in the onset of intestinal inflammation remains elusive.

**Methods:**

To close that knowledge gap, we performed a comprehensive transcriptomic analysis of different patient cohorts. Moreover, we investigated the therapeutic efficacy of Sema3B *in vivo*.

**Results:**

Our findings revealed that the expression of *SEMA3B* was downregulated in IBD patients compared with healthy controls. Similarly, non-responder UC patients to infliximab showed reduced transcript levels of that class-3 semaphorin before receiving the treatment. Unfortunately, the administration of recombinant Sema3B to mice subjected to DSS-acute colitis did not modify the course of the disease.

**Conclusions:**

Therefore, SEMA3B appears to be an interesting biomarker in the context of intestinal inflammation, which deserves further validation in larger cohorts. Nonetheless, based on our *in vivo* results, the implication of this factor in the development of colitis seems to be minimal.

## Background

Ulcerative colitis (UC) is a chronic and relapsing inflammatory disease that affects the colon and rectum mucosa. The etiopathogenesis of this disorder is not completely understood, but it results from an aberrant immune response to changes in the gut microbiota in genetically susceptible individuals. The worldwide increase in the incidence and prevalence of UC, along with the associated morbidity, leads to significant socioeconomic challenges. In addition, the current therapeutic armamentarium, including anti-inflammatory or immunosuppressive drugs, corticoids, biologic agents and small molecules, aims to alleviate UC symptoms, but they do not offer a cure and are linked to treatment resistance and potential serious side effects. Therefore, there is a need to search new classification criteria and safer drugs to combat intestinal inflammation.

Semaphorins constitute a large family of proteins initially related to neural system development. Class-3 semaphorins (sema3A-3G) contain semaphorin, plexin-semaphorin-integrin (PSI), and immunoglobulin-like (Ig) domains, but differ from the other proteins within this family by the presence of a basic C-terminal domain. Also, contrary to other semaphorins, sema3A- G are secreted proteins. Class-3 semaphorins are ligands of the Type-A plexins (PlexinA1-A4) transmembrane receptors, which function as signal transducers, and the neuropilin (NRP) scaffold receptors, NRP-1 and -2. Apart from their role in controlling axon guidance development, it is now evident that these proteins regulate other relevant biological processes underlying inflammatory diseases, such as angiogenesis, apoptosis, cell migration, or immune function. For instance, the administration of SEMA3A to mice prevented renal damage in a mouse model of systemic lupus erythematosus (1) and reduced disease severity in arthritis models (2, 3). Similarly, reduced expression of SEMA3E has been observed in severe asthmatic patients (4), and its genetic ablation in mice led to more severe airway inflammation (5). In line with this, *SEMA3E* expression was found downregulated in active UC patients, and *Sema3e^—/—^* mice showed exacerbated colitis symptoms and severity as compared to their wild-type counterparts (6). Additionally, we observed decreased SEMA3B levels in the synovial tissue of rheumatoid arthritis (RA) patients in comparison to arthralgia and undifferentiated arthritis patients (7, 8), and treatment with this class-3 semaphorin reduced fibroblast-like synoviocytes spontaneous migration (7) and induced a proresolutive phenotype in RA monocyte-derived macrophages (8). Moreover, *Sema3b^—/—^* mice showed higher severity of K/BxN serum-induced arthritis, and the exogenous administration of recombinant SEMA3B had a therapeutic effect on that same arthritis preclinical model (9). Collectively, these findings suggest that class-3 semaphorins can regulate uncontrolled inflammatory responses in different inflammatory immune-mediated diseases (IMID). However, it is not yet known whether SEMA3B is associated with UC patients or whether it contributes to the onset of intestinal inflammation. To address those gaps, we performed a comprehensive transcriptomic analysis using multiple publicly available datasets combined with *in vivo* experiments, which revealed that SEMA3B might be more relevant as a biomarker than for its functional involvement in the development of intestinal inflammation.

## Methods

### Transcriptomic databases

We collected gene expression data from the Gene Expression Omnibus (GEO) database (http://www.ncbi.nlm.nih.gov/geo/). The microarray gene sets GSE38713 (10) and GSE59071 (11) contain expression profiles of colon biopsies obtained from non-IBD and UC patients with different disease activity after receiving therapy with immunosuppressants and/or corticosteroids (active disease was defined by endoscopic Mayo subscore ≥2). The microarray datasets GSE16879 (12) and GSE12251 (13) include gene expression profiles of colon biopsies from patients with UC before initiating anti-TNF (infliximab) treatment (response was defined based on endoscopic and histologic healing at 4–6 or 8 weeks, respectively, after initiation of treatment). The microarray gene set GSE73661 (14) contains the expression profile of colon biopsies obtained from UC patients before starting treatment with infliximab, as well as UC patients before initiating anti-integrin α4β7 (vedolizumab) therapy (response was defined as endoscopic mucosal healing Mayo endoscopic subscore 0 or 1, assessed at 4–6 weeks for infliximab, and 6, 12 and 52 weeks for vedolizumab). The microarray gene sets GSE92415 (15) and GSE212849 (16) include gene expression profiles of colon biopsies from patients with UC before initiating golimumab treatment (clinical response was defined at 6 weeks based on a decrease in the Mayo score ≥30% and ≥ 3 points or from baseline). The RNAseq gene set GSE191328 (17) includes expression profiles of blood cells obtained from patients with UC before starting treatment with vedolizumab and infliximab (response was defined as partial Mayo score ≤2). The RNAseq gene expression dataset E-MTAB-7845 encompasses the expression profiles of colon biopsies from UC patients before initiating vedolizumab therapy (18) (response was defined as Mayo endoscopic subscore ≤1). This dataset was obtained from the European Bioinformatics Institute (EMBL-EBI) database (https://www.ebi.ac.uk/biostudies/arrayexpress/studies/). Volcano plots were generated with Glimma by plotting the log2 fold changes and corresponding -log10 p values obtained from the differential expression analyses performed with limma (linear models for microarray data).

### DSS-induced acute colitis

All animal experiments were conducted according to the Spanish animal experimentation legislation: Law 32/2007 7^th^ November and Royal Decree 51/2013 1^st^ February. All procedures were approved by the local Ethics Committee (Comité de Ética de Experimentación Animal (CEEA) de los centros usuarios de animales de experimentación de la USC en el Campus de Santiago and Health Research Institute of Santiago de Compostela Órgano Habilitado) and with the registration number: 15012/2024/012. Also, this study is in accordance with ARRIVE guidelines.

Ten to twelve-week-old female C57BL/6 mice (n=18) (provided by The Center for Experimental Biomedicine of the University of Santiago de Compostela, registry number: ES150780292901) were subjected to acute colitis by administering 1.5% of dextran sodium sulphate (DSS) (MP Biomedicals, CA, USA; Cat. no. 9011-18-1) in their drinking water for 7 days. From day 0, the mice received intraperitoneal injections of either saline, IgG2A (10 µg/day/mouse), or mouse recombinant SEMA3B (10 µg/day/mouse). The Disease Activity Index (DAI) was determined by assessing the mice daily for visual appearance, behaviour, activity, pain, percentage weight loss, stool consistency and blood in the stool. Each parameter was scored as follows: 0 (normal), 1 (very moderate), 2 (moderate), 3 (medium), 4 (elevated). The DAI was represented as the sum of all the scores. On the last day of the experiment, the animals were euthanized by carbon dioxide inhalation followed by cervical dislocation for sample collection. No anaesthesia agent was used.

### Histology

Colon tissue sections were fixed in 4% formalin for 24 hours, followed by a dehydration process using a series of different ethyl alcohol concentrations (ranging from 70% to 100%) before being embedded in paraffin wax. 4 μm thick sections were cut using a rotary microtome (Zeiss, Germany) and incubated at 60°C for 1 hour. For hematoxylin and eosin (H&E) staining, tissue sections were deparaffinized with Histo Clear (National Diagnostics, GA, USA; Cat. no. HS-200), rehydrated using a graded series of ethyl alcohol (ranging from 100% to 70%) and then stained with hematoxylin (Agilent, CA, USA; Cat. no. CS70030-2) for 10 minutes, followed by 2 seconds differentiating step with 1% HCL in ethanol. Subsequently, the sections were further stained for 10 seconds with 1% eosin (pH 5.2) (Agilent, CA, USA; Cat. no. CS70130-2), dehydrated with a series of ethyl alcohol (ranging from 70% to 100%), and finally mounted with Pertex (HistoLab, Sweden; Cat. no. 00801). The processed samples were examined under a light microscope Zeiss Axio Vert a1 (Zeiss, Germany), and images were taken at 20X magnification.

The most distal part of the colon was selected for histological analysis, and two independent investigators, blinded to the type of treatment, conducted the histological examination.

Inflammatory infiltration and epithelial damage scoring were performed as follows:

Epithelial damage: normal morphology = 0; loss of goblet cells = 1; loss of goblet cells in large areas = 2; loss of crypts = 3; loss of crypts in large areas = 4.

Infiltration: no infiltrate = 0; infiltrate around crypt basis = 1; infiltrate reaching to lamina muscularis mucosae = 2; extensive infiltration reaching the lamina muscularis mucosae and thickening of the mucosa with abundant oedema = 3; infiltration of the lamina submucosa = 4.

### Flow cytometry

We used flow cytometry to analyse the numbers of lamina propria lymphocytes (LPLs). First, we obtained a single-cell suspension from LPLs by cutting a colon segment into approximately 0.5 mm3 pieces. Then, we incubated the pieces with HBSS (Sigma-Aldrich, MO, USA; Cat no. H6136) plus EDTA (2 mM) (Sigma-Aldrich, MO, USA; Cat no. E5134) for 15 minutes at 37°C in an orbital shaker. After washing with HBSS, colon pieces were incubated in HBSS plus EDTA (2 mM) buffer for 30 minutes at 37°C in an orbital shaker. The remaining colon pieces were digested using a digestion buffer containing dispase (0.6 mg/mL) (Abnova, Taiwan; Cat. no. P5318) and collagenase IV (0.4 mg/mL) (Sigma-Aldrich, MO, USA; Cat no. 11088858001) for 20 minutes at 37°C in an orbital shaker. The digested colon tissues were finally homogenized using a syringe and 18G needle (BD, NJ, USA; Cat. no. 304622) and filtered through a 70 μm cell strainer (Avantor, PA, USA; Cat. no. 732-2758).

For surface staining, we incubated the LPLs with the following panel of fluorescent-labelled antibodies (Biolegend, CA, USA) for 20 minutes: PerCP anti-CD45 (Cat. no. 103129); Spark Blue 550 anti-CD3 (Cat. no. 100259); PE-Fire 640 anti-F4/80 (Cat. no. 157319); PerCP-Cy5.5 anti-MHCII (Cat. no. 107626); PE anti-CD11c (Cat. no. 117308); PE-Cy7 anti-Ly6C (Cat. no. 128018); PE-Dazzle594 anti-Ly6G (Cat. no. 127647); Zombie Green (Cat. no. 423111) was used as viability dye.

We also used eBioscience™ Annexin V Apoptosis Detection Kit FITC (Thermo Fisher Scientific, MA, USA; Cat. no. 88-8005-74) to analyse the viability/apoptosis of cells, according to the manufacturer’s protocol.

The cells were analyzed on a CYTEK Cytometer (Cytek Biosciences, Fremont, CA, USA), and OMIQ software was used to process and represent flow cytometry data.

### RNA isolation and real-time reverse transcription–polymerase chain reaction

The colonic tissue samples were homogenized in NZYol (NZYTech, Portugal; Cat. no. MB18501) using a TissueLyser II (QIAGEN, The Netherlands) for 2 minutes at 30 Hz. After homogenization, the RNA was extracted using the E.Z.N.A Total RNA Kit (Omega Bio-tek, GA, USA; Cat. no. R6834-02) according to the manufacturer’s instructions. The quantity and quality of the total RNA were evaluated using the NanoDrop ND-1000 spectrophotometer (NanoDrop Technologies, DE, USA). Reverse transcription-quantitative real-time polymerase chain reaction (RT-qPCR) was performed using a Power SYBR™ Green RNA-to-CT™ 1-Step Kit (Applied Biosystems, MA, USA; Cat. no. 4389986) with 10 ng of total RNA per sample. The data were calculated using the comparative (2^−ΔΔCt^) method and Quantstudio Design and Analysis Software v.1.5.3 (Applied Biosystems, MA, USA). Control cells were used as a reference and were assigned an arbitrary number of 1. The specific PCR primers used are included in **Table 1** (Thermo Fisher Scientific, Waltham, MA, USA), and Sema3g (NM_001025379.1) (QIAGEN, The Netherlands; Cat. No. 330001). It is noteworthy that the normalization of gene expression levels was performed in relation to β-Actin. Melting curves were generated to ensure a single gene-specific peak, and no-template controls were included for each run and each set of primers to detect unspecific amplifications.

**Table 1:** List of primers used for RT-qPCR.

Gene	Forward	Reverse
Sema3b	AAGGTGCAGCAACTGCTTTG	CAGGCAGTAAAGGGCGAAGA
Sema3c	GAGGGCTCTACCCTTCCATC	GCCTTCAGCTTGCCATAGTC
Sema3f	TGGAACCGAACACACCTGTA	GGACATTTGCCTTTTCCTGA
Il6	ACAACCACGGCCTTCCCTACTT	CACGATTTCCCAGAGAACATGTG
Il1b	AACCTGCTGGTGTGTGACGTTC	CAGCACGAGGCTTTTTTGTTGT
Tnf-α	CTACTCCCAGGTTCTCTTCAA	GCAGAGAGGAGGTTGACTTTC
Ccl2 (MCP1)	CCACTCACCTGCTGCTACTCAT	TGGTGATCCTCTTGTAGCCCTCC
Cxcl2 (MIP-2α)	TCCAGAGCTTGAGTGTGACG	GCAAACTTTTTGACCGCCCT
Cxcl5 (ENA-78)	GCTGGCATTTCTGTTGCTGT	TAGCTATGACTTCCACCGTAGG
Cxcl1	AATGAGCTGCGCTGTCAGTG	TGAGGGCAACACCTTCAAGC
Cxcr1	AATCTGTTGTGGCTTCACCCA	GCTATCTTCCGCCAGGCATAT
Plxna1	CTTCTGGACTGGGCTCTGAC	TAGAGGGTGGCTCTGAGCAT
Plxna2	AACCTGTCTGTGGTTCTGCTC	TCCAGTCACGATTCTCAGAGT
Plxna3	CTGCCCTCTGGAGAGTTACG	GCCAACACAGGACATACACG
Plxna4	ACAGGGCACATTTATTTGGGG	CACTTGGGGTTGTCCTCATCT
Nrp1	GGAGCTACTGGGCTGTGAAG	ACCGTATGTCGGGAACTCTG
Nrp2	ATTCAGAAAGCTGGGGGTTT	GAGCCTCAAATCAGCCAAAG

### Protein extraction and western blot analysis

The colonic tissue was homogenizated in cold RIPA buffer [all purchased in Sigma-Aldrich, St. Louis, MO, USA; 10 mM Tris–HCl (Cat. no. T6066 and Cat. no. 30721), pH 7.5, 5 mM EDTA, 150 mM NaCl (Cat. no. S5886), 30 mM sodium pyrophosphate (Cat. no. 221368), 50 mM sodium fluoride (Cat. no. S7920), 1 mM sodium orthovanadate (Cat. No. S6508), 0.5% Triton X-100 (Cat. No. 93443), 1 mM PMSF (Cat. no. P7626)] supplemented with protease inhibitor cocktail (Thermo Fisher Scientific, MA, USA; Cat. no. 78438) using a TissueLyser II. Next, whole lysates were centrifuged at 13.000 rpm for 20 minutes. The lysates were separated on 12% Sodium dodecyl-sulfate polyacrylamide gel electrophoresis (SDS-PAGE). Subsequently, proteins were transferred to a nitrocellulose membrane (Cytiva, MA, USA; Cat. no. 10600003). The membranes were blocked in 5% BSA (Sigma-Aldrich, MO, USA; Cat. no. A7906) for 1 hour. Immunoblots were incubated overnight with the specific antibody against Claudin-4 (Cell Signaling Technology, MA, USA; Cat.no 94478), diluted 1:1000. The appropriate anti-rabbit (Cytiva, MA, USA; Cat. no. 12352203) secondary antibody diluted 1:2000 was incubated for 1 hour. The immune complexes were detected by using the Chemiluminescence HRP (Reagent for Horseradish Peroxidase) SuperSignal West Pico PLUS (Thermo Fisher Scientific, MA, USA; Cat. no. 15669364). Finally, to ensure equal protein loading, the membrane was incubated with the anti-GAPDH antibody diluted 1:5000 (Sigma-Aldrich, St. Louis, MO, USA; Cat. no. G9545). The images were captured with ChemiDoc MP Imaging System (Bio-Rad Laboratories, CA, USA) and analyzed with ImageJ Software.

### Myeloperoxidase activity

Colon specimens, approximately 0.5 cm long, were homogenized in phosphate buffer (50mM, pH 6.0) and 0.5% hexadecyltrimethylammonium bromide (Sigma-Aldrich, MO, USA; Cat.no. H5882) using a TissueLyser II. Following three freeze-and-thaw cycles, the resulting supernatant was combined with 0.02% dianisidine (Sigma-Aldrich, St. Louis, MO, USA; Cat.no. D9143) in phosphate buffer (50mM, pH 6.0), and 0.0005% H_2_O_2_ (Sigma-Aldrich, St. Louis, MO, USA; Cat. no. H1009). MPO activity, expressed as arbitrary units, was determined as the average absorbance at 460 nm per gram of protein content.

### Statistical analysis

ROC curves were performed with R (version 4.3.1). For statistical analysis, we employed GraphPad Prism 8.0.1 software (Graph-Pad Software, Boston, MA, USA). Quantitative data are presented as the mean ± standard error of the mean (SEM) within the different treatment groups. Two-group comparisons were conducted using a two-tailed Student t-test. Multigroup comparisons were performed using one-way ANOVA followed by the Bonferroni multiple-comparison test after the application of the appropriate normality test. A p-value of <0.05 was considered statistically significant. Correlations were performed using the Spearman’s rank correlation coefficient (r).

## Results

### Colonic *SEMA3B* expression is decreased in inflammatory bowel disease patients

**Figure 1:**
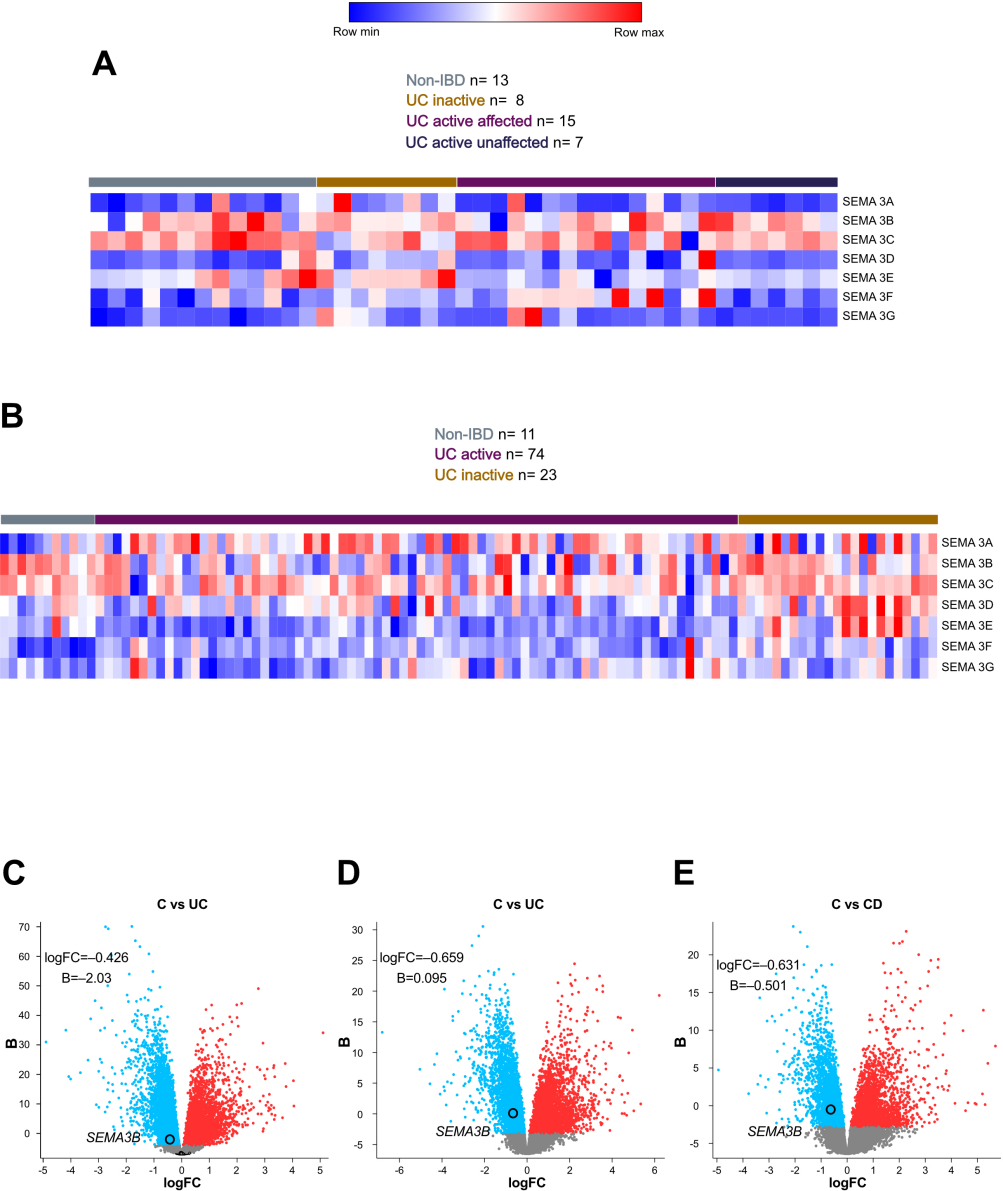
*SEMA3B* expression is altered in inflammatory bowel disease patients. Heatmap encompassed the relative expression of the interrogated transcripts in colon biopsies from the indicated groups of patients. The results were retrieved from the GEO microarray **(A)** GSE38713 (Non-IBD n=13; UC inactive n=8; UC active affected n=15; UC active unaffected n=7) and **(B)** GSE59071 (Non-IBD n=11; UC inactive n=23; UC active n=74). Volcano plots showing the differentially expressed genes in human colon biopsies of the GEO microarray datasets **(C)** GSE73661 (control; n=12; UC; n=44), and **(D, E)** GSE16879 (control; n=12; UC; n=24; CD; n=37) in the indicated groups. The upregulated and downregulated transcripts are represented in red and blue dots, respectively. B=log–odds; logFC=Fold Change.

First, we analysed the expression of all class-3 semaphorins in the colon using two independent microarray datasets. We found that most semaphorins did not exhibit any variation among non-IBD, active UC or inactive UC patients (**Figures 1A, B**; **Supplementary Figure S5**). Only *SEMA3B* expression appeared to be downregulated in active UC patients, a trend that was more evident in **Figure 1B**. To confirm this observation, we also studied the transcript levels of *SEMA3B* in another three cohorts. Interestingly, the expression of this class-3 semaphorin was significantly decreased in UC and CD patients than in healthy controls (**Figure 1C**), opposite to that observed for the IBD gold standard biomarker calprotectin (**Supplementary Figures S1A, B**).

### SEMA3B-related receptors are dysregulated in UC patients

**Figure 2:**
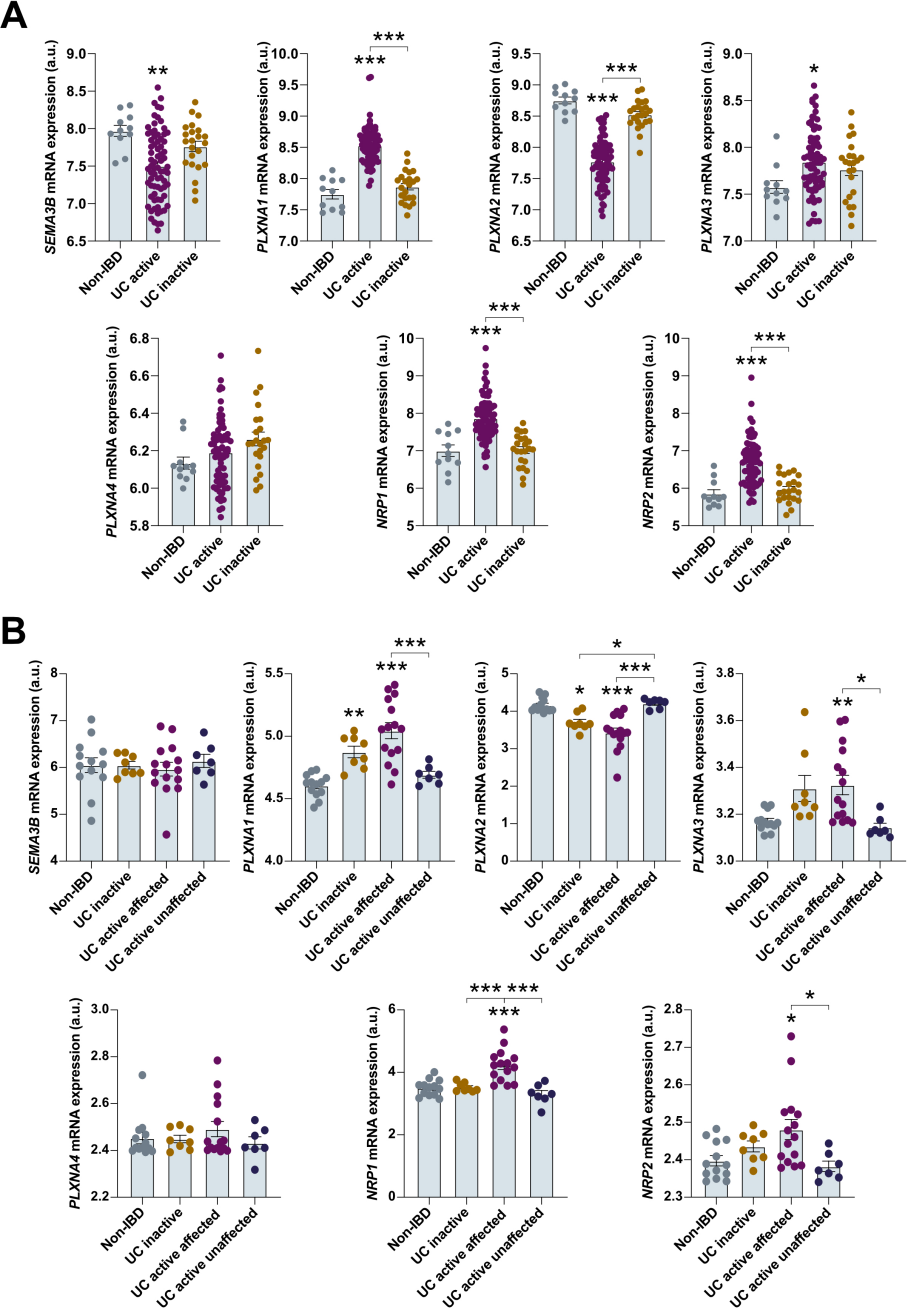
The expression of SEMA3B-associated receptors is altered in ulcerative colitis patients. Relative expression of the interrogated transcripts in colon biopsies from the indicated groups of patients. The results were retrieved from the GEO microarray dataset **(A)** GSE59071 (Non-IBD n=11; UC inactive n=23; UC active n=74) and **(B)** GSE38713 (Non-IBD n=13; UC inactive n=8; UC active affected n=15; UC active unaffected n=7). *P ≤ 0.05; **P ≤ 0.01; ***P ≤ 0.001 relative to Non-IBD, unless indicated different.

In addition to the previously mentioned downregulation of *SEMA3B* mentioned above, we also detected that the expression of the SEMA3B-associated transmembrane receptors *PLXNA1–2* and *3* was dysregulated in UC patients (**Figures 2A, B**). Specifically, *PLXNA1* and *PLXNA3* were upregulated in active UC patients, while *PLXNA2* exhibited decreased expression in individuals with active disease (**Figures 2A, B**). We observed a similar pattern of upregulation for the scaffold receptors *NRP1* and *NRP2* (**Figures 2A, B**).

### *SEMA3B*, *PLXNA2*, and *NRP2* expression is altered in infliximab non-responder patients at baseline

**Figure 3:**
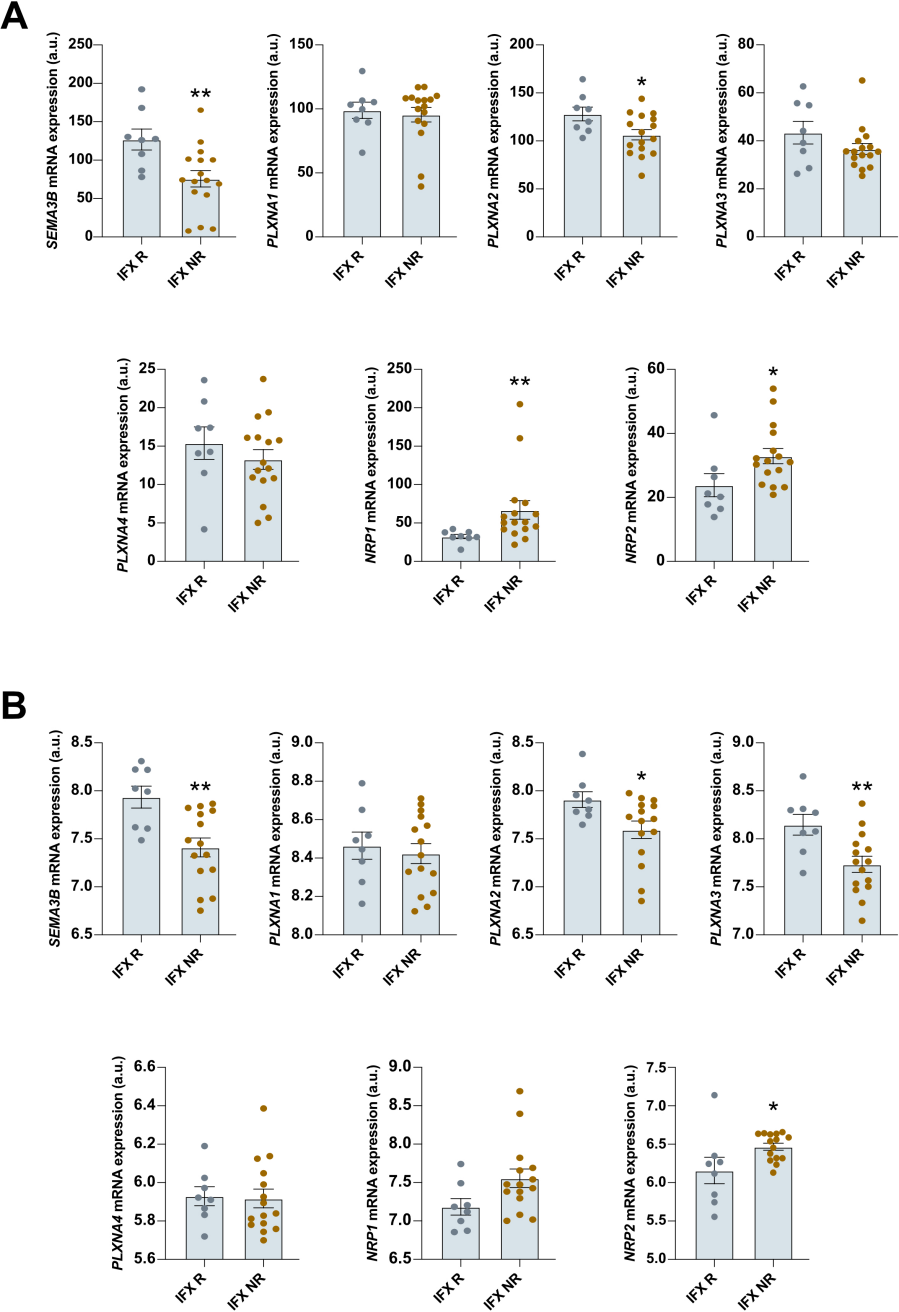
The expression of *SEMA3B*, *PLXNA2* and *NRP2* is dysregulated in ulcerative colitis patients before infliximab therapy. Relative expression of the interrogated transcripts in colon biopsies from the indicated groups of patients. The results were retrieved from the GEO microarray dataset **(A)** GSE16879 (Responder n=8; Nonresponder n=16) and **(B)** GSE73661 (Responder n=8; Nonresponder n=15). IFX R=infliximab responder; IFX NR=infliximab nonresponder. *P ≤ 0.05; **P ≤ 0.01 relative to IFX R.

Next, we investigated whether SEMA3B and its associated receptors can serve as predictors of biologic therapy response. First, as shown in **Figure 3**, significantly downregulated colonic expression before treatment of *SEMA3B* and *PLXNA2*, as well as upregulated expression of *NRP2*, was associated with the lack of response to infliximab in two different cohorts of UC patients. *PLXNA3* and *NRP1* also showed dysregulated expression at baseline in non-responder patients. However, the differences only reached statistical significance in one of the datasets studied. *PLXNA1* and *PLXNA4* did not present any significant variation related to infliximab treatment response (**Figures 3A, B**). Second, to evaluate the reliability of these biomarkers in predicting infliximab response, we conducted a receiver operating characteristic (ROC) analysis. However, we found low discriminative power for *SEMA3B* expression (ROC AUC = 51.6%) and for the combination of *SEMA3B*, *PLXNA2* and *NRP2* expression (ROC AUC = 49.9%).

**Figure 4:**
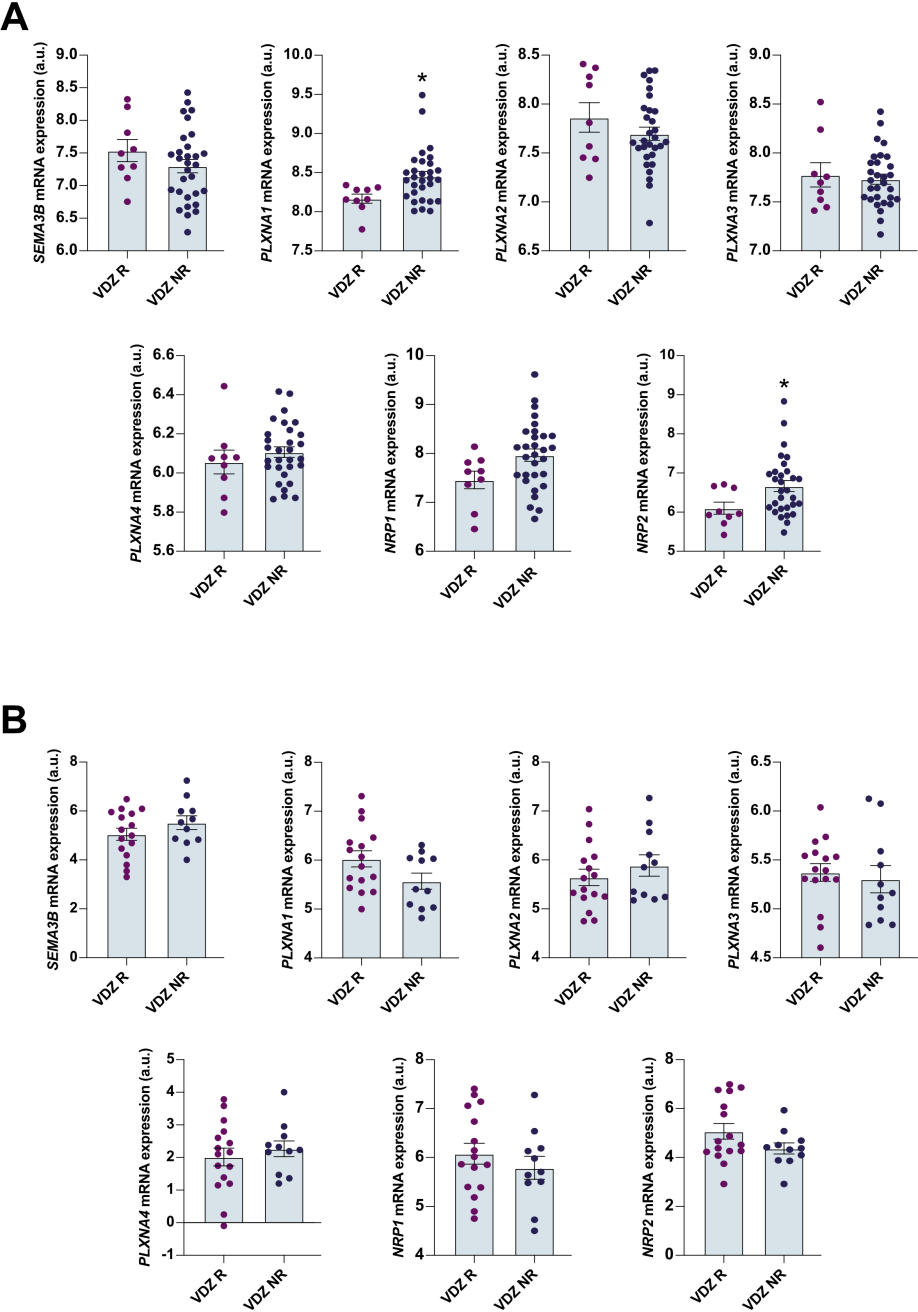
The expression of *SEMA3B* and its associated receptor is not altered in ulcerative colitis patients before vedolizumab therapy. Relative expression of the interrogated transcripts in colon biopsies from the indicated groups of patients. The results were retrieved from the GEO microarray dataset **(A)** GSE73661 (Responder n=9; Nonresponder n=30) and **(B)** E-MTAB-7845 (Responder n=16; Nonresponder n=11). VDZ R=vedolizumab responder; VDZ NR=vedolizumab nonresponder. *P ≤ 0.05 relative to VDZ R.

To corroborate whether this finding also applies to other anti-TNF therapies, we performed a similar analysis on colon biopsies from UC patients starting golimumab therapy. Contrary to the observations made with infliximab, patients starting golimumab did not present significant variations in *SEMA3B* expression across two different patient cohorts (**Supplementary Figure S2**). The SEMA3B-associated receptors were not correlated to golimumab responsiveness at baseline, except for *PLXNA4*, *NRP1* and *NRP2*, which showed variations in only one of the datasets analysed (**Supplementary Figure S2**). We also carried out a similar analysis using data from UC patients undergoing treatment with vedolizumab, which has a different mechanism of action. Notably, the baseline expression of *SEMA3B* and *PLXNA2* did not differ between responders and non-responders (**Figures 4A, B**). *NRP2* transcript levels were also increased in non-responder patients before vedolizumab treatment (**Figure 4A**), but this result was not consistent in a different cohort (**Figure 4B**). The same was observed for *PLXNA1* (**Figures 4A, B**). In general, the expression of *SEMA3B* and its associated receptors showed less variation in patients initiating vedolizumab therapy compared to infliximab. To note, *SEMA3B* expression showed more consistency in these particular patient cohorts than the postulated anti-TNF therapy response biomarker TREM-1 (19) (**Supplementary Figures S1C–D**).

### Recombinant Sema3B does not affect intestinal inflammation *in vivo*

**Figure 5:**
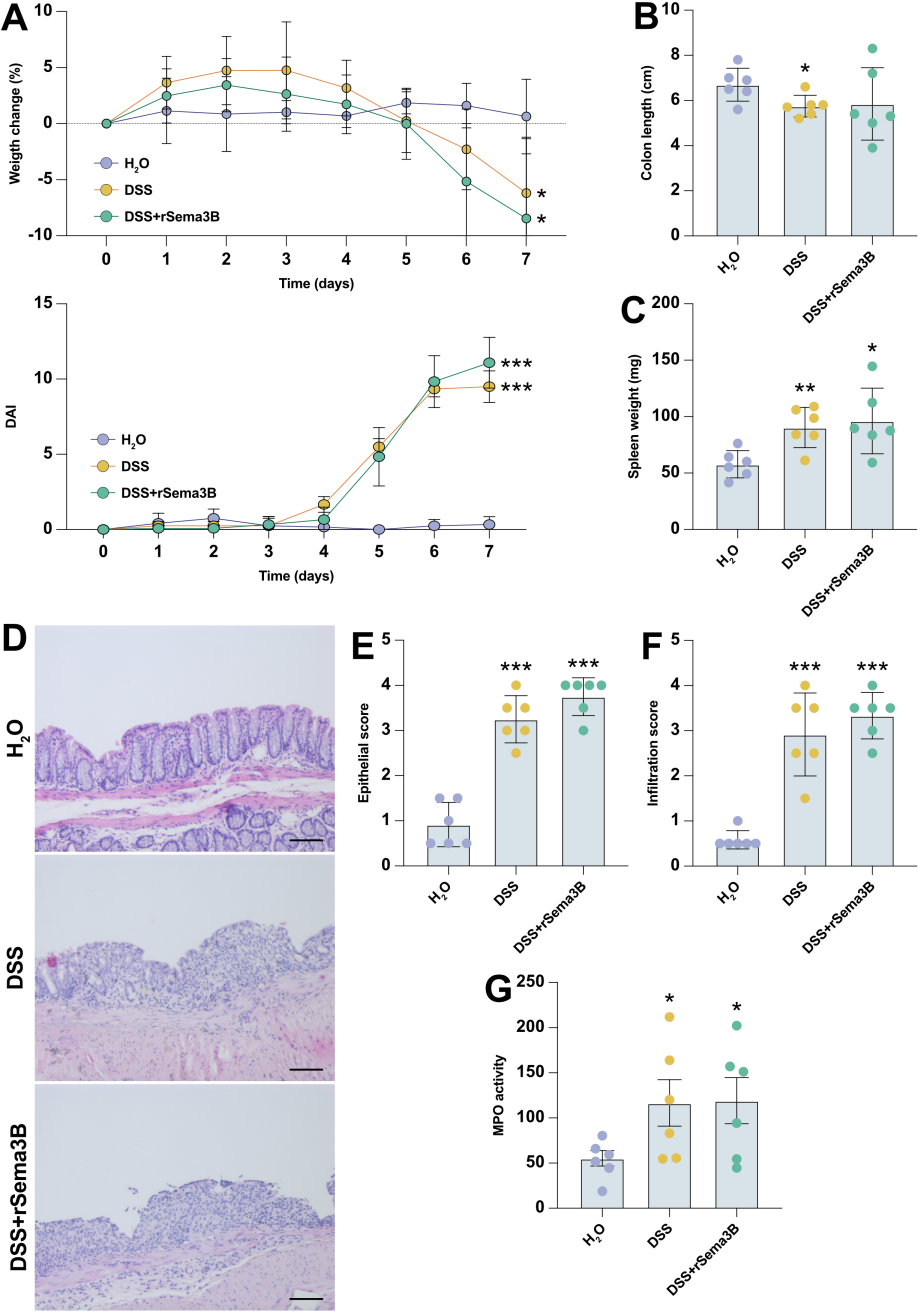
The administration of recombinant Sema3B does not affect the development of intestinal inflammation *in vivo*. Percentage of weight loss **(A)**, representation of the disease activity index (DAI) **(A)**, colon length **(B)** and spleen weight **(C)** in mice of the indicated groups during DSS-acute colitis. Representative histology images **(D)**, epithelial damage scoring **(E)**, infiltration scoring **(F)** and MPO activity **(G)** in mice of the indicated groups during DSS-acute colitis. *P ≤ 0.05; **P ≤ 0.01; ***P ≤ 0.001 relative to control mice (H_2_O). Scale bar: 100 μm.

Transcriptomic data revealed that *SEMA3B* and its receptors were dysregulated in UC patients with active disease and showed an association with the response to infliximab therapy in these patients. This suggested that SEMA3B could represent a novel intestinal pathophysiological pathway that warranted further investigation. Consequently, we aimed to explore whether SEMA3B signalling represents a novel putative therapeutic approach to alleviate intestinal inflammation *in vivo*. To study the functional involvement of SEMA3B in the development of colitis, we administered intraperitoneally recombinant Sema3B (rSema3B) to mice subjected to DSS-induced acute colitis. Unfortunately, rSema3B treatment failed to reverse the weight loss or mitigate the disease activity index (DAI) caused by the DSS (**Figure 5A**). Similarly, the colon length did not differ significantly at the end of the experiment between DSS and DSS+rSema3B mice (**Figure 5B**). We also measured the spleen weight as an indicator of systemic inflammation, but adding rSema3B to mice did not modify the increase in spleen weight elicited by DSS (**Figure 5C**). Histological analysis of mouse colon sections confirmed those results. As shown in **Figure 5D**, the administration of DSS induced strong epithelial barrier damage, epithelial hyperplasia and the infiltration of immune cells into the submucosa compared with the control mice. However, the administration of rSema3B could not prevent the loss of the normal architecture of the colon mucosa induced by the DSS. Accordingly, epithelial and infiltration subscores of both DSS and DSS+rSema3B were significantly increased compared with control mice (**Figures 5E, F**), and MPO activity showed the same pattern (**Figure 5G**).

**Figure 6:**
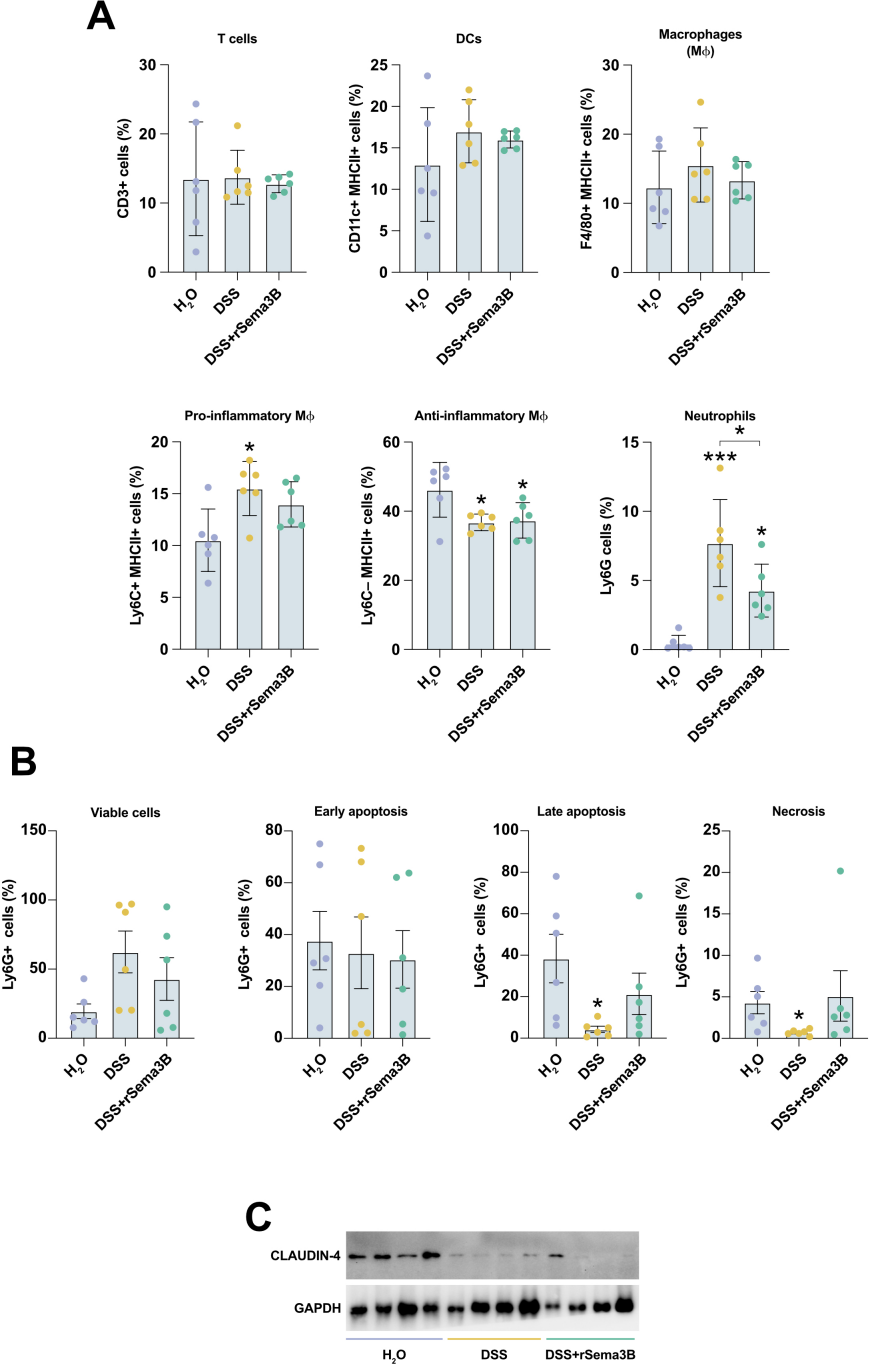
The administration of recombinant Sema3B to colitic mice decreases the number of neutrophils in the lamina propria. **(A)** Frequencies of the indicated cell populations analysed by flow cytometry in LPLs of the indicated groups of animals. **(B)** Frequencies of viable, apoptotic and necrotic neutrophils detected by flow cytometry in LPLs of the indicated groups of animals. **(C)** Protein expression detected by western blot of the interrogated proteins in the colon tissue of the indicated groups of animals. *P ² 0.05; ***P ² 0.001 relative to control mice (H2O), unless indicated different.

We also analysed the inflammatory cell infiltrate in the colon lamina propria, and as expected, the relative numbers of most of the cell populations did not differ between DSS and DSS+rSema3B groups (**Figure 6A**). Only the neutrophil percentage was significantly reduced after the administration of rSema3B (**Figure 6A**). Further analysis of neutrophil composition revealed that although not significant, rSema3B-treated mice had less viable, and more apoptotic and necrotic neutrophils compared to DSS-treated mice (**Figure 6B**).

### Recombinant SEMA3B does not significantly affect colon epithelial barrier and inflammation *in vivo*

Finally, we explored the impact of Sema3B on the maintenance of the colon epithelial barrier. The administration of DSS significantly decreased the expression of the tight junction protein CLAUDIN-4 compared to untreated mice, and as expected, rSema3B did not influence the protein expression of CLAUDIN-4 (**Figure 6C**).

**Figure 7:**
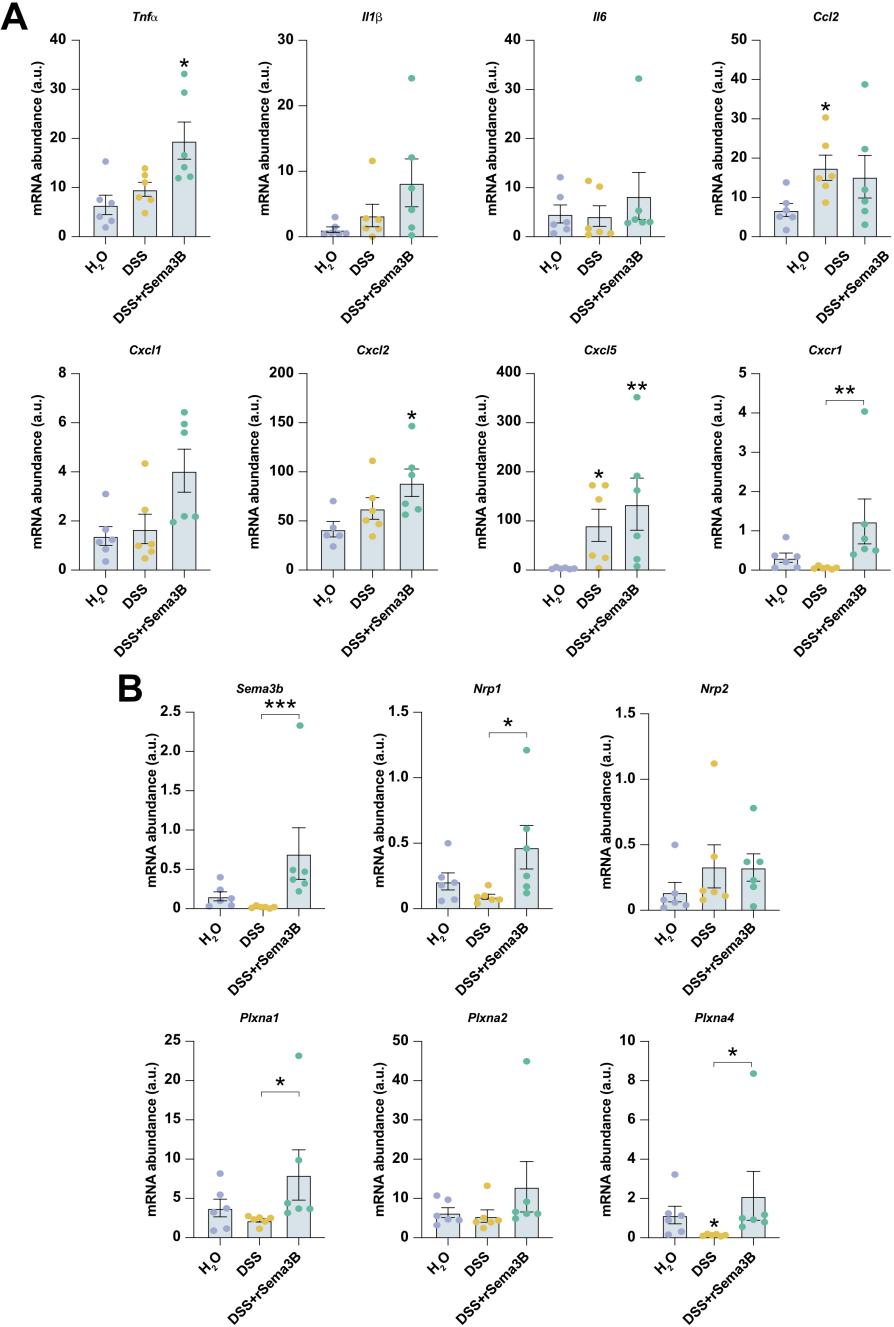
The administration of recombinant Sema3B to colitic mice does not significantly modulate inflammatory factors expression. Relative expression of the interrogated transcripts in colon tissue of the indicated groups of animals **(A, B)**. *P ² 0.05; **P ² 0.01; ***P ² 0.001 relative to control mice (H2O), unless stated different.

Similarly, rSema3B administration induced an increased tendency in the expression of *Tnfa*, *Il1b*, *Cxcl1*, *Cxcl2* and *Cxcl5*, and a significant upregulation of *Cxcr1* compared to DSS mice (**Figure 7A**). To note, mice treated with rSema3B exhibited increased transcript levels of *Sema3b*, *Nrp1* and *Plxna2* compared to the DSS group (**Figure 7B**), as well as *Sema3c*, *Sema3f* and *Sema3g* (**Supplementary Figure S3**).

## Discussion

In this study, we found a dysregulated expression of *SEMA3B* and related receptors in the colon tissue of patients with UC, which correlates with disease activity. This down-regulation is in line with previous works showing a reduced SEMA3B expression in several conditions, including different types of cancer (20), idiopathic lung fibrosis (21) and rheumatoid arthritis (7, 8). Additionally, the expression levels of PLXNA1–3 and NRP1–2 were significantly associated with UC activity. This suggests that using this combination of semaphorin and associated receptors, alongside a gold standard biomarker like calprotectin, could enhance the sensitivity of evaluating disease activity in these patients. Although the lack of data regarding PLXNA and NRP receptors in IBD underscores the need to validate these findings in other datasets and/or larger patient cohorts.

SEMA3B has been postulated as a promising biomarker of disease progression in the above-mentioned conditions, but to our knowledge, this is the first study indicating that SEMA3B may be a useful predictive biomarker of response to therapy. Our expression data clearly indicate an association between SEMA3B levels and the responsiveness to infliximab therapy, although ROC analysis did not demonstrate significant predictive value. This low discriminative power can result from the limited sample size or a general lack of predictive utility of these biomarkers. We cannot rule out the possibility either that reduced SEMA3B expression in non-responders is due to differences in TNF levels. In fact, TNF reduces SEMA3B expression in synovial fibroblasts and endothelial cells (7, 22), and we found a negative correlation trend between SEMA3B and TNF expression, and a positive correlation with the TNF receptor TNFRSF1A in the analysed datasets (**Supplementary Figure S4**). Therefore, further studies are required to draw more definitive conclusions. In any case, the combination of low *SEMA3B* and *PLXNA2* expression, along with high *NRP2* transcriptional levels, presents an intriguing biomarker value. While single-specific biomarkers like TREM-1 have notable advantages in terms of clinical application due to their simplicity, the transcriptional complexity among IBD patients suggests that using gene signatures composed of multiple genes may serve as a more effective predictive tool. Different gene signatures with high accuracy in predicting response to anti-TNF therapy have been reported (23, 24). Notably, TREM-1 and the other four genes were consistently dysregulated in non-responder patients to IFX (25), highlighting the utility of specific combinations of biomarkers. Moreover, our data show variability in TREM-1 expression across different patient cohorts, emphasising the importance of employing biomarkers from a different nature (e.g. TREM-1 and semaphorin-related genes) in combination to increase the predictive power for therapy responses. It is important to note that neither *SEMA3B*, *PLXNA2*, nor *NRP2* were differentially expressed in responder *versus* non-responder patients to golimumab or VDZ therapy. This finding underscores the specificity of these biomarkers for infliximab rather than their association with clinical remission. Interestingly, we have reported a negative correlation between TNF expression and SEMA3B expression in the synovial tissue of patients with RA, and that TNF reduces, in RA fibroblast-like synoviocytes and endothelial cells, the expression of SEMA3B and HOXA5, a transcription factor responsible for SEMA3B expression (7, 22). Altogether, these results demonstrate the interplay between SEMA3B and TNF.

We also analysed the functional implications of SEMA3B. The administration of recombinant Sema3B to mice subjected to DSS-induced acute colitis showed no therapeutic efficacy, contrasting with our previous observations in the K/BxN serum-induced arthritis model (9). Both K/BxN serum-induced arthritis and DSS-induced acute colitis models heavily rely on neutrophils and macrophages (26, 27). Transcriptomic data from the K/BxN serum-induced arthritis model revealed that *Sema3b^—/—^* mice exhibited impaired neutrophil functions (9), consistent with the decreased numbers of these granulocytes shown in this study. However, Sema3B appeared to affect macrophage function more efficiently in the context of inflammatory arthritis than during colitis. This suggests that possibly, the lack of that dual impairment did not allow SEMA3B to modulate the course or the severity of the intestinal inflammation. Also, the inability of Sema3B to modulate the expression of proinflammatory mediators may have contributed to its lack of disease-modifying activity, as opposed to the observed effect after the administration of recombinant Sema3E to DSS-induced colitis mice, where intestinal inflammation was mitigated along with the reduction in the colonic levels of Il6, Tnfα, or Il1β (6) Another plausible explanation is due to the upregulation of *PlexinA1* and *Nrp1* observed in rSema3B-treated mice. PlexinA1 and NRP1 are receptors of other class 3 semaphorins, which may have inflammatory effects. In fact, it has been reported that Sema3G plays pathogenic roles in mice models of arthritis (28), while Sema3C induces a macrophage pro-inflammatory phenotype in a mouse model of neuroinflammation (29). In line with this, Sema3F promotes neutrophil retention in the inflamed tissue and poor resolution of inflammation in murine lung injury models (30). Importantly, *Sema3c*, *Sema3f* and *Sema3g* expression was also up-regulated in rSema3B-treated mice (**Supplementary Figure S1**). Then, these data suggest that the potential anti-inflammatory role of Sema3B may be compensated by the inflammatory effects of Sema3C and Sema3G. The opposite effect of class 3 semaphorins has been observed in other pathological processes since Sema3B plays a protective role in pulmonary fibrosis (21), while Sema3C exacerbates liver fibrosis (31).

Another significant finding of our study is the reduction in neutrophil numbers in the colonic lamina propria caused by Sema3B treatment. While other semaphorins have been shown to regulate neutrophil migration and chemotaxis (32, 33), this is the first report showing that a member of this family of proteins can affect neutrophil apoptosis *in vivo*. However, the modulation of neutrophil apoptosis did not modify the severity of the DSS model and therefore the functional consequences must be elucidated in further work. The role of neutrophils in colitis remains controversial. Studies have shown that depleting this cell type can exacerbate colitis symptoms in rats treated with dinitrobenzene sulfonic acid (DNBS) or in mice undergoing T cell transfer colitis (34). However, opposite results were observed in rats after the administration of DSS (35). These discrepancies may be attributed to variations in experimental models, differences between animal species and the factors involved in cell depletion. Moreover, we must consider the complex interactions between neutrophils, other immune cells, and their roles in bacterial containment (36). This dual role of neutrophils might explain why a decrease in their numbers does not always correlate with an overall improvement in inflammatory symptoms.

This study has several limitations. First, the Sema3B serum levels were not measured. This analysis should be performed in future studies, in order to validate the use of SEMA3B as a biomarker or response to therapy using a less invasive technique. Second, we used a single concentration of rSema3B (10 µg/day/mouse), based on a previous experiment from our group (9). Then, we cannot rule out the possibility that this concentration does not work in the DSS model. Finally, the *in vivo* model focused on the role of SEMA3B in the initiation of the disease, but this protein might be involved in the resolution of the inflammation. In fact, a recent study showing that Sema3B promotes a pro-resolving phenotype in macrophages from patients with rheumatoid arthritis supports this hypothesis (8).

Altogether, our work postulates SEMA3B as a potential biomarker in the context of intestinal inflammation, although its implication in the pathogenesis of the disease appears to be limited.

## Data Availability

The raw data supporting the conclusions of this article will be made available by the authors, without undue reservation.

## Supplementary material

Supplementary Figure 1 Determination of the expression of calprotectin and TREM-1 in ulcerative colitis patients. Relative expression of the interrogated transcripts in colon biopsies from the indicated groups of patients. The results were retrieved from the GEO microarray dataset **(A)** GSE59071 (Non-IBD n=11; UC inactive n=23; UC active n=74); **(B)** GSE38713 (Non-IBD n=13; UC inactive n=8; UC active affected n=15; UC active unaffected n=7); **(C)** GSE16879 (Responder n=8; Nonresponder n=16); **(D)** GSE73661 (Responder n=8; Nonresponder n=15); **(E)** GSE73661 (Responder n=9; Nonresponder n=30) and **(F)** E-MTAB-7845 (Responder n=16; Nonresponder n=11). *, P ≤ 0.05; **, P ≤ 0.01; ***, P ≤ 0.001 relative to Non-IBD, IFX R or VDZ R, unless indicated different.

Supplementary Figure 2 The expression of *SEMA3B*, *PLXNA2* and *NRP2* is not dysregulated in ulcerative colitis patients before golimumab therapy. Relative expression of the interrogated transcripts in colon biopsies from the indicated groups of patients. The results were retrieved from the GEO microarray dataset (A) GSE92415 (Responder n=32; Nonresponder n=27) and (B) GSE212849 (Responder n=21; Nonresponder n=63). GLM R=golimumab responder; GLM NR=golimumab nonresponder. *, P ≤ 0.05; **, P ≤ 0.01 relative to GLM R.

Supplementary Figure 3 The administration of recombinant Sema3B to colitic mice induces the expression of other members of the class-3 semaphorins. Relative expression of the interrogated transcripts in the colon tissue of the indicated groups of animals (A, B, and C).

Supplementary Figure 4 *SEMA3B* expression correlates with the expression of the TNF receptor TNFRSF1A in patients with UC treated with infliximab. Correlations of the indicated genes in colon biopsies from the indicated groups of patients. The results were retrieved from the GEO microarray dataset (A) GSE16879 (Responder n=8; Nonresponder n=16) and (B) GSE73661 (Responder n=8; Nonresponder n=15). IFX R=infliximab responder; IFX NR=infliximab nonresponder. *, P ≤ 0.05; ***, P ≤ 0.001.

Supplementary Figure 5 Representation of the expression of SEMA3B in percentage across different patient groups. Heatmap encompassed the percentage of expression of the interrogated transcripts in colon biopsies from the indicated groups of patients. The results were retrieved from the GEO microarray **(A)** GSE38713 (Non-IBD n=13; UC inactive n=8; UC active affected n=15; UC active unaffected n=7) and **(B)** GSE59071 (Non-IBD n=11; UC inactive n=23; UC active n=74). Data are shown as the percentage respect to the mean value of the Non-IBD group for each individual gene.
