# Supplementary material for: SEMA3B is associated with disease activity and infliximab response in IBD patients but does not contribute to the development of intestinal inflammation in vivo
Source: Front Immunol. 2026 Feb 4;17:1677130. doi: 10.3389/fimmu.2026.1677130 (PMC12913132; doi:10.3389/fimmu.2026.1677130)
Supplement: Supplementary Figure 1 — Determination of the expression of calprotectin and TREM-1 in ulcerative colitis patients. Relative expression of the interrogated transcripts in colon biopsies from the indicated groups of patients. The results were retrieved from the GEO microarray dataset (A) GSE59071 (Non-IBD n=11; UC inactive n=23; UC active n=74); (B) GSE38713 (Non-IBD n=13; UC inactive n=8; UC active affected n=15; UC active unaffected n=7); (C) GSE16879 (Responder n=8; Nonresponder n=16); (D) GSE73661 (Responder n=8; Nonresponder n=15); (E) GSE73661 (Responder n=9; Nonresponder n=30) and (F) E-MTAB-7845 (Responder n=16; Nonresponder n=11). *, P ≤ 0.05; **, P ≤ 0.01; ***, P ≤ 0.001 relative to Non-IBD, IFX R or VDZ R, unless indicated different. [file DataSheet1.docx]

Supplementary Material

#

# Supplementary Figures and Tables

## Supplementary Figures


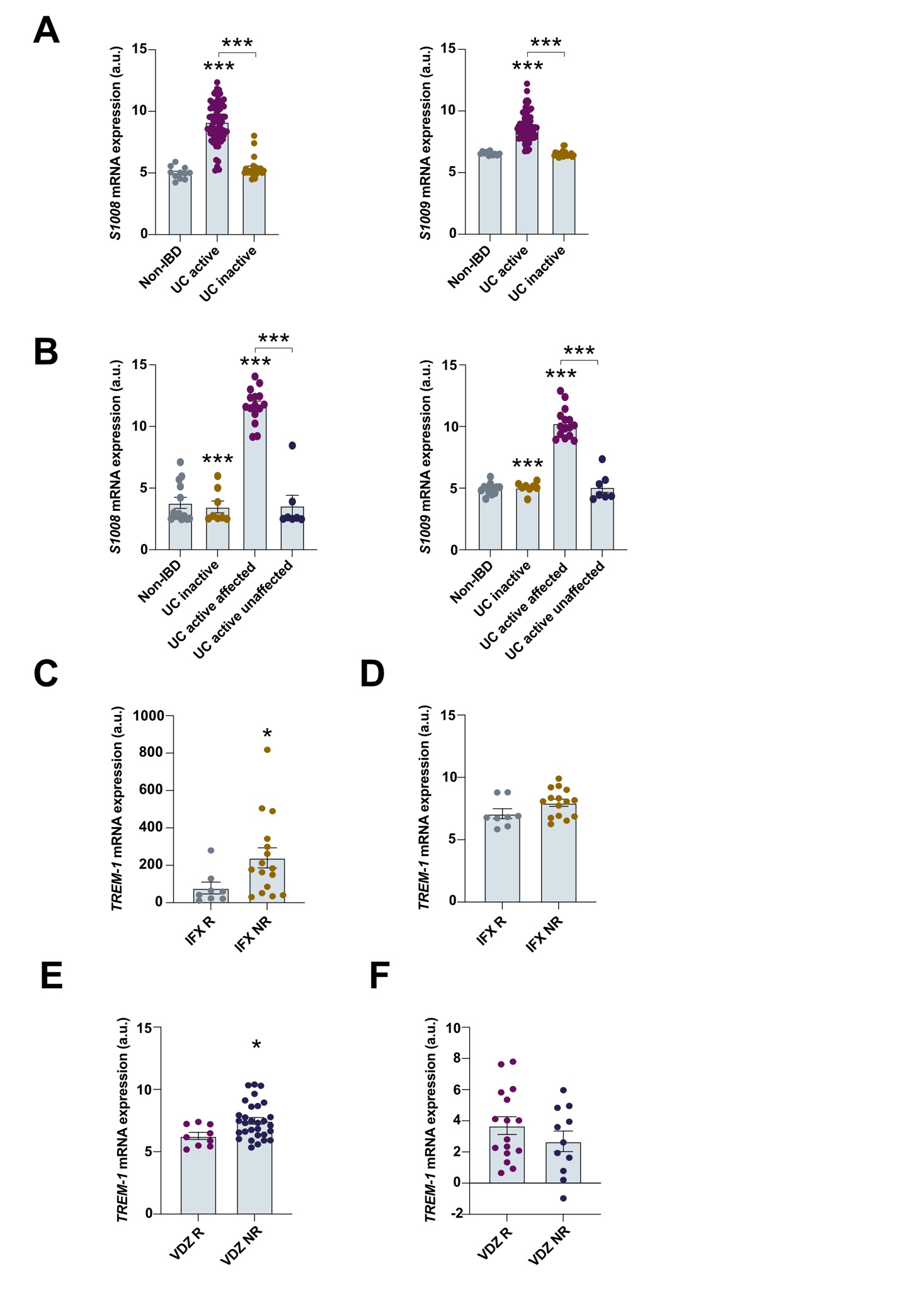


**Supplementary Figure 1.** **Determination of the expression of calprotectin and TREM-1 in ulcerative colitis patients.** Relative expression of the interrogated transcripts in colon biopsies from the indicated groups of patients. The results were retrieved from the GEO microarray dataset **(A)** GSE59071 (Non-IBD n=11; UC inactive n=23; UC active n=74); **(B)** GSE38713 (Non-IBD n=13; UC inactive n=8; UC active affected n=15; UC active unaffected n=7); **(C)** GSE16879 (Responder n=8; Nonresponder n=16); **(D)** GSE73661 (Responder n=8; Nonresponder n=15); **(E)** GSE73661 (Responder n=9; Nonresponder n=30) and **(F)** E-MTAB-7845 (Responder n=16; Nonresponder n=11). *, P ≤ 0.05; **, P ≤ 0.01; ***, P ≤ 0.001 relative to Non-IBD, IFX R or VDZ R, unless indicated different.

**
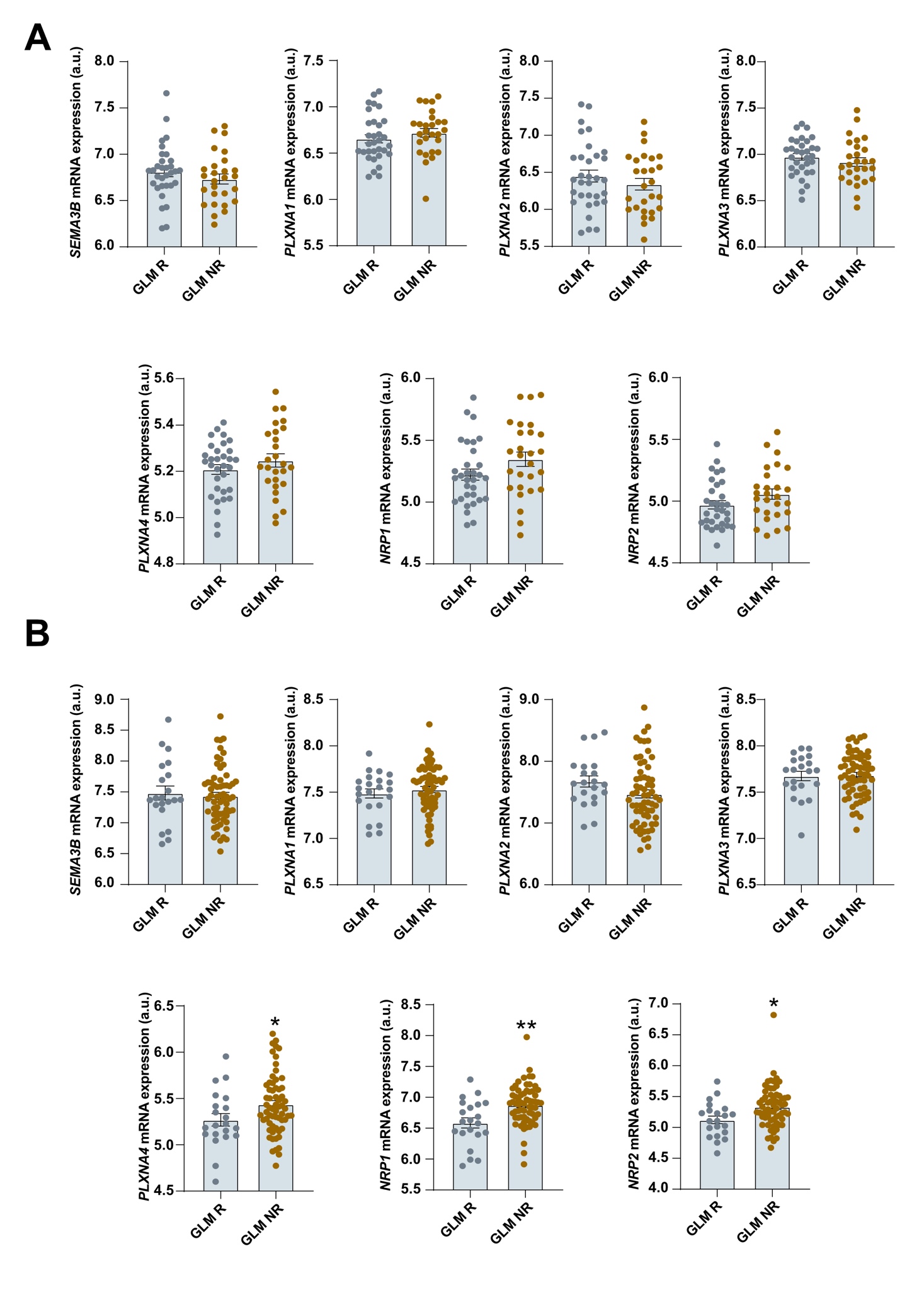
**

**Supplementary Figure 2.** **The expression of *SEMA3B*, *PLXNA2* and *NRP2* is not dysregulated in ulcerative colitis patients before golimumab therapy.** Relative expression of the interrogated transcripts in colon biopsies from the indicated groups of patients. The results were retrieved from the GEO microarray dataset **(A)** GSE92415 (Responder n=32; Nonresponder n=27) and **(B)** GSE212849 (Responder n=21; Nonresponder n=63). GLM R=golimumab responder; GLM NR=golimumab nonresponder. *, P ≤ 0.05; **, P ≤ 0.01 relative to GLM R.

**Supplementary Figure 3. The administration of recombinant Sema3B to colitic mice induces the expression of other members of the class-3 semaphorins.** Relative expression of the interrogated transcripts in the colon tissue of the indicated groups of animals **(A, B, and C)**.


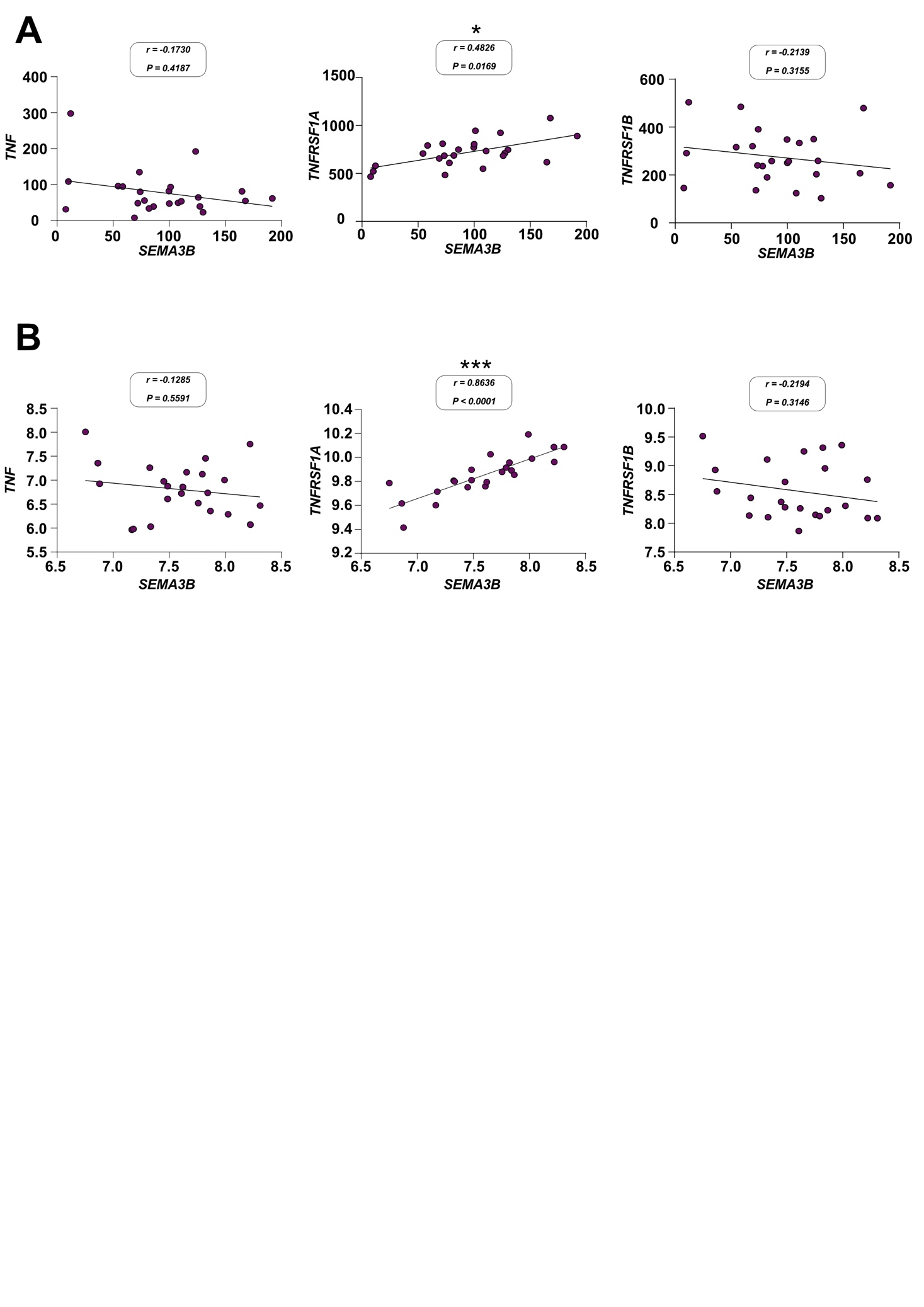


**Supplementary Figure 4. *SEMA3B* expression correlates with the expression of the TNF receptor TNFRSF1A in patients with UC treated with infliximab.** Correlations of the indicated genes in colon biopsies from the indicated groups of patients. The results were retrieved from the GEO microarray dataset **(A)** GSE16879 (Responder n=8; Nonresponder n=16) and **(B)** GSE73661 (Responder n=8; Nonresponder n=15). IFX R=infliximab responder; IFX NR=infliximab nonresponder. *, P ≤ 0.05; ***, P ≤ 0.001.

**Supplementary figure 5. Representation of the expression of SEMA3B in percentage across different patient groups.** Heatmap encompassed the percentage of expression of the interrogated transcripts in colon biopsies from the indicated groups of patients. The results were retrieved from the GEO microarray (A) GSE38713 (Non-IBD n=13; UC inactive n=8; UC active affected n=15; UC active unaffected n=7) and (B) GSE59071 (Non-IBD n=11; UC inactive n=23; UC active n=74). Data are shown as the percentage respect to the mean value of the Non-IBD group for each individual gene.
